# Comprehensive analysis of the association of *EGF*R, *CALM3* and *SMARCD1* gene polymorphisms with BMD in Caucasian women

**DOI:** 10.1371/journal.pone.0112358

**Published:** 2014-11-14

**Authors:** Qiu-Hong Zhou, Lan-Juan Zhao, Ping Wang, Rhamee Badr, Xiao-Jing Xu, Feng-Xiao Bu, Joan Lappe, Robert Recker, Yu Zhou, An Ye, Bo-Ting Zhou

**Affiliations:** 1 Department of Endocrinology, Xiangya Hospital, Central South University, Changsha Hunan, 410008, China; 2 Osteoporosis Research Center, Creighton University Medical Center, Creighton University, 601 N 30th ST, Suite 4820, Omaha, Nebraska, 68131, United States of America; 3 Department of Biostatistics & Bioinformatics, School of Public Health and Tropical Medicine, Tulane University, New Orleans, Louisiana, 70112, United States of America; 4 Department of Pharmacy, Xiangya Hospital, Central South University, Changsha Hunan, 410008, China; 5 Tulane University School of Medicine, New Orleans, Louisiana, 70112, United States of America; Centers for Disease Control and Prevention, United States of America

## Abstract

**Summary:**

Three genes, including *EGFR* (epidermal growth factor receptor), *CALM3* (calmodulin 3, calcium-modulated protein 3) and *SMARCD1* (SWI/SNF-related matrix-associated actin-dependent regulator of chromatin subfamily d member 1), play different roles in bone and/or fat metabolism in Caucasian women. In this population-based investigation of 870 unrelated postmenopausal Caucasian women, *CALM3* polymorphisms were significantly associated with femoral neck bone mineral density (FNK BMD), hip BMD and spine BMD. Age and tobacco status also affected BMD levels and were therefore corrected for in our statistical analysis.

**Introduction:**

*EGFR*, *CALM3* and *SMARCD1* play roles in bone and/or fat metabolism. However, the correlations between the polymorphisms of these three genes and body composition levels, including BMD, remain to be determined.

**Materials and Methods:**

A population-based investigation of 870 white women was conducted. Forty-four SNPs (single nucleotide polymorphisms) in *EGFR*, *CALM3* and *SMARCD1* were chosen by the software, including those of potential functional importance. The candidate SNPs were genotyped by the KASPar assay for an association analysis with body composition levels. The correlation analysis was assessed by the Pearson's product-moment correlation coefficient and Spearman rank-order correlation tests, and the family-wise error was corrected using the Wald test implemented in PLINK.

**Results:**

The SNP rs12461917 in the 3′-flanking region of the *CALM3* gene was significantly associated with FNK BMD (P = 0.001), hip BMD (P<0.001) and spine BMD (P = 0.001); rs11083838 in the 5′-flanking region of *CALM3* gene was associated with spine BMD (P = 0.009). After adjusting for multiple comparisons, rs12461917 remained significant (P-adjusted  = 0.033 for FNK BMD, P-adjusted  = 0.006 for hip BMD and P-adjusted  = 0.018 for spine BMD).

**Conclusions:**

Our data show that polymorphisms of the *CALM3* gene in Caucasian women may contribute to variations in the BMD of the hip, spine and femoral neck.

## Introduction

Osteoporosis is a common progressive bone disease that is characterized by decreased bone mineral density (BMD) and is known to increase the risk of fractures [1]. The disequilibration of bone resorption by osteoclasts and bone formation by osteoblasts underlies the pathogenesis of osteoporosis [2]. At-risk populations for primary osteoporosis include elderly and postmenopausal women in particular because BMD is known to decrease with age and its rate of decline is very hormonally sensitive [3]. Estrogen may exert anti-resorptive effects on bone in part by stimulating estrogen receptors and osteoprotegerin expression in osteoblasts [4]. Estrogen is one of many proteins that are involved in the pathogenesis of osteoporosis. Age and external factors, such as smoking, body weight and race, also influence BMD [5], [6], and more recently, investigators have found that genetic factors play important roles in the pathogenesis of osteoporosis [7]. Twin and familial studies have indicated that 60–85% of BMD variance is genetically determined [8]. Candidate gene association studies have found several polymorphisms that are associated with BMD, bone loss and osteoporotic fractures, such as those of the vitamin D receptor, collagen type 1 α1, estrogen receptor α, lipoprotein receptor related protein 5 and TGF-β1 [7]. The three novel target genes that were under investigation in the present study included *EGFR*, *CALM3* and *SMARCD1*, all of which play known roles in bone or embryonic-related fat metabolism. The purpose of our study was to investigate the associations between polymorphisms of these genes and body composition levels, including spine, hip and FNK BMD, total body fat (TBF), trunk fat (TF), percentage of total body fat (PTBF), percentage of trunk fat (PTF), total body lean mass (TBL) and trunk lean mass (TL). To the best of our knowledge, this is the first study to investigate these relationships.

*EGFR*-deficient mice have been previously demonstrated to exhibit delayed primary endochondral ossification due to defective osteoclast recruitment [9]. EGFR has also more recently been shown to play an anabolic role in bone metabolism in vivo [10]. An *EGFR* dominant negative allele, Wa5, that was introduced into transgenic mice led to a nearly complete knockdown of *EGFR* activity in preosteoblasts/osteoblast lineage cells. These mice exhibited remarkable decreases in their tibial trabecular bone masses, alterations in their tibial microarchitectures and decreases in osteoblast numbers and mineralization activities. Similarly, the administration of an *EGFR* inhibitor into wild-type mice caused a significant reduction in trabecular bone volume [10]. Subsequent studies have found that *EGFR* signaling promotes the proliferation and survival of osteoprogenitors by increasing early growth response 2 expression [11].

Another molecule, calmodulin (CaM), which is primarily encoded by the calmodulin 3 gene (*CALM3*), regulates EGFR activity by directly interacting with the CaM binding domain of the receptor [12], [13] and has been shown to be a critical regulator of osteoclast differentiation, functional bone resorption and osteoclast apoptosis [14]. During the process of active bone resorption, CaM expression is increased and concentrated at the ruffled border. The interaction of CaM with the FAS death receptor has also been implicated in osteoclast apoptosis, and signal transduction pathways involving CaM and its downstream effectors, such as calcineurin and CaM kinase II(CAMKII), have been shown to regulate osteoclastogenesis [14]. Through its direct interaction with EGFR or its other effectors, or both, CALM3 is also intimately involved in bone metabolism, and hence, we set out to further explore the role of CALM3 and the related gene *EGFR* in the context of their correlations with BMD and other body composition metrics.

Bone and fat tissues share the same embryonic origin of mesenchymal stem cells (MSC) [15]. Fat mass is a significant determinant of BMD [16], although the mechanism behind this correlation remains unclear. Although there is no evidence in support of the bone response to static loads, several authors have suggested that fat mass acts by increasing the muscle-mediated skeletal dynamic load, thereby causing bone remodeling and changes in BMD [17]. Other authors have reported that hormonal influences (estrone production by fat tissues, leptin, etc.) underlie the fat-mediated strengthening/remodeling of bone [17]. BAF60a [SWI/SNF-related matrix-associated actin-dependent regulator of chromatin subfamily D member 1(SMARCD1)] acts as a molecular link between SWItch/Sucrose NonFermentable (SWI/SNF) chromatin-remodeling complexes and hepatic lipid metabolism that has been shown to regulate lipid homeostasis [18]. In mouse models, the adenovirus-mediated expression of SMARCD1 was shown to induce peroxisomal and mitochondrial fat oxidation and lower hepatic triglyceride levels [18]. Because SMARCD1 is involved in lipid metabolism, it may also correlate with body composition (total body fat, trunk fat, etc.) and perhaps even BMD because of the correlation of BMD with fat body mass. Furthermore, using modified yeast hybrid screens, SMARCD1 has been shown to interact with the VDR heterodimer complex, alluding to a more direct route by which SMARCD1 may influence bone metabolism [19].

Tobacco usage contributes to many chronic diseases, including cardiovascular disease, chronic obstructive pulmonary disease (COPD), cancer and osteoporosis [20], [21], [22], [23]. A meta-analysis was previously performed to assess the effects of cigarette smoking on BMD. Pooled data across 86 studies and 40,753 patients demonstrated that smokers had significantly reduced bone masses compared with nonsmokers at all sites, with an average of 1/10 standard deviation deficit for the combined sites. Deficits that were associated with the hips of smokers were even more pronounced, 1/3 standard deviation lower than those of nonsmokers. At the hip, the BMD of current smokers was one-third of a SD less than that of never smokers. Moreover, smoking increases the lifetime risk of developing a vertebral fracture by 13% in women and 32% in men [24].

Other studies have echoed this same trend [25]. Additionally, postmenopausal women may be particularly at risk for smoking-related bone loss. For example, another previous meta-analysis found that although premenopausal bone densities were similar in female smokers and nonsmokers, postmenopausal bone loss was greater in current smokers than nonsmokers with bone density decreases of an additional 2% for every 10-year increase in age [26]. Some studies have suggested that lower BMD in smokers may in part be attributable to their lower body weights and fat masses [27]; however, evidence has indicated that bone mass differences remain significant after controlling for body weight and age [24]. Thus, other mechanism may be responsible. In fact, recent literature has supported the presence of molecular mechanisms that play important roles in smoking-related bone loss. For example, a recent study found that smoke carcinogens cause bone loss through the aryl hydrocarbon receptor and the induction of Cyp1 enzymes [28]. Smoking may also influence the expression of many genes and biomarkers of immune B cells [20], [21]. Immune B cells are generated in the bone marrow and are known to play significant roles in bone metabolism and secrete many cytokines and factors that regulate osteoclastogenesis and ostoblastogenesis [29], [30]. Consequently, we hypothesized that smoking would impact body composition levels. Because we found this to be true in our study population, we accounted for smoking and age in our statistical analysis of the effects of the polymorphisms of our three target genes on BMD and body composition.

## Materials and Methods

### Subjects

We recruited 1179 unrelated postmenopausal Caucasian women, including smokers and non-smokers, who were over 55 years of age from a 9 county rural area in the midwestern U.S. A total of 870 qualified subjects were retained after applying the exclusion criteria. Details of the recruitment and subsequent exclusion criteria were previously reported [31], [32]. All participants were generally healthy. In the current study, the subjects' primary phenotypes, which were measured in the prior study and included weight, height, body mass index (BMI), BMD at the spine, hip and FNK and other body composition data, such as TBF, TF, TBL and TL. Details of these phenotypes and their measurement were previously reported [32]. All subjects provided written informed consent, and the Institutional Review Board at Creighton University approved the project. We applied for clinical admission with the Clinical Trials.gov Identifier: NCT00352170.

### Single nucleotide polymorphism (SNP) selection and genotyping

Tag SNPs of the three genes were selected from the software program Haploview version 4.2 (http://www.broad.mit.edu/haploview/haploview, accessed on September 18th, 2009) with minor allele frequencies (MAF) >10% in the HapMap CEU (western European ancestry) population. Based on the HapMap database (http://www.hapmap.org, release 28, on August 16th 2010), the tag SNPs were selected with the following thresholds: pairwise r^2^ (r^2^≥0.8) and haplotype R^2^ (R^2^≥0.8). In addition to these tag SNPs, we also chose other SNPs of previously reported potential functional importance, specifically SNPs in the promoters, 3′-UTRs (untranslated regions) and exons of the target genes. All of these SNPs were authenticated using the HapMap (http://www.hapmap.org) and NCBI (http://www.ncbi.nlm.nih.gov/SNP/) databases. DNA was extracted from peripheral blood with the Gentra Puregene Blood Kit (Qiagen Inc.,Valencia, California) according to the manufacturer's protocol. The KASPar assay was used to genotype the target SNPs with 20 µg/ml of the DNA samples (KBioscience; http://www.kbioscience.co.uk). All of the selected SNPs were genotyped. Of the 870 patients, 846 were successfully genotyped according to a threshold call rate of ≥95%. The genotype call rates ranged from 97.6–99.8%, and the average call rate was 99.2%.

### Statistical analyses

All analyses were conducted using the Statistics Package for Social Science, version 18.0 (SPSS Inc., Chicago, Illinois). The Hardy-Weinberg equilibrium (HWE) of the genotypic frequencies among the subjects was assessed. Pearson's product-moment correlation was used as the parametric test and Spearman rank-order correlation was performed as the non-parametric test. These correlation analyses were carried out to elucidate the potential effects of covariates on body compositions, including age and smoking status. A linear regression test was used to adjust body compositions according to the significant covariates, and the adjusted compositions were used for the data analyses.

Because repeated testing can produce false-positive results, the results were corrected and the *P* values were adjusted accordingly. To control for the family-wise error rate, a permutation procedure using the Wald test that was implemented in PLINK (version 1.0.7) [33], which is an open-source tool set, was conducted. The adjusted *P* value for the SNP was denoted as the proportion of n/5,000. The test of significance was two-tailed, and alpha was set at 0.05.

## Results

### Basic characteristics of participants

Of all 870 qualified subjects, 846 were successfully genotyped and their levels of BMD were detected, and the TBF, TBL and PTBF of 744 subjects in addition to the TF, TL and PTF of 725 were successfully tested. **Table 1** lists the basic characteristics of the participants. The subjects had a mean age of 60.8±9.1 (SD) years, mean height of 163.1±6.2 cm, mean weight of 74.9±14.4 kg and mean BMI of 28.1±5.2 kg/m^2^. Their mean spine BMD, hip BMD and FNK BMD were 0.97±0.16 g/cm^2^, 0.90±0.13 g/cm^2^ and 0.75±0.12 g/cm^2^, respectively. Of the 846 successfully genotyped participants, 336 were smokers and 510 were non-smokers. Mean TBF and TF were 29.9±9.56 and 14.0±5.25, respectively. TBL and TL were 44.4±6.02 and 22.0±2.97, respectively. Percentages of TBF and TF were 39.2±6.56 and 37.8±7.60, respectively.

**Table 1:** Basic characteristics of the participants at baseline.

Variables	
Age (years, n = 846)	60.8±9.1
Height (cm, n = 846)	163±6.2
Weight (kg, n = 846)	74.9±14.4
BMI (kg/m^2^, n = 846)	28.1±5.2
Tobacco use (yes/no, n = 846)	336/510
Femoral neck BMD (g/cm^2^, n = 846)	0.751±0.123
Spine BMD (g/cm^2^, n = 846)	0.970±0.160
Hip BMD (g/cm^2^, n = 846)	0.902±0.131
Total body fat (n = 744)	29.9±9.57
Total body lean mass (n = 744)	44.4±6.02
Percentage of total body fat (n = 744)	39.2±6.56
Trunk fat (n = 725)	14.0±5.25
Trunk lean mass (n = 725)	22.0±2.97
Percentage of trunk fat (n = 725)	37.8±7.60

Values represent the means±SD.

BMI: body mass index, calculated as weight divided by the square of height.

BMD: bone mineral density.

Percentages of fat (including total body fat and trunk fat) were calculated as fat divided by the total fat and lean mass, multiplied by 100.

### Basic characteristics of 3 candidate genes and 44 SNPs selected from the three genes

**Table 2** shows the basic characteristics of the three candidate genes. *EGFR*, *CALM3* and *SMARCD1* are located on chromosomes 7p12.1, 19q13.2 and 12q13.12, respectively. **Table 3** lists the basic characteristics of the 44 target SNPs of the 3 genes. All SNPs were consistent with Hardy–Weinberg equilibrium. The MAF ranged from 10–50%, and the average MAF was 30.8% in the candidate cohort of 846 subjects.

**Table 2:** Basic characteristics of the three candidate genes.

Official symbol	Official full name	Reference for gene function	Gene type	Location	Length (kb)	Number of exons	Candidate SNPs
*CALM3*	calmodulin 3 (phosphorylase kinase, delta)	Toutenhoofd et al.(1998)	Protein coding	19q13.2-q13.3	9.5	6	5
*SMARCD1*	SWI/SNF-related matrix-associated actin-dependent regulator of chromatin subfamily d member 1	Li et al.(2008)	Protein coding	12q13-q14	15.7	12	4
*EGFR*	epidermal growth factor receptor	Chandra et al.(2013)	Protein coding	7p12	18.8	28	35

**Table 3:** Basic characteristics of the 44 selected SNPs of the three candidate genes (n = 846).

Gene	SNP	Locus	Location (bp)	Allele	Function	MAF	HWE
*CALM3*	rs11083838	5′ near gene	19356457	C/T	promoter	10.4	0.699
*CALM3*	rs7259810	promoter	19371646	T/C		42.5	0.395
*CALM3*	rs4380146	intron 1	19374852	G/T		32.8	0.477
*CALM3*	rs10113	3′-UTR	19380866	C/T	3′-UTR	47.8	1.000
*CALM3*	rs12461917	3′ near gene	19385620	A/C		12.4	0.768
*SMARCD1*	rs11169270	intron 6	12626338	A/G		33.6	0.558
*SMARCD1*	rs7139363	intron 10	12631853	A/G		20.7	0.512
*SMARCD1*	rs836172	intron 10	12633405	G/C		43	0.334
*SMARCD1*	rs836177	intron 11	12635127	G/A		43	0.284
*EGFR*	rs6969537	promoter	4671787	A/G		14.2	0.974
*EGFR*	rs4947963	intron 1	4677784	C/T		35.1	0.447
*EGFR*	rs763317	intron 1	4684666	A/G		47.9	0.997
*EGFR*	rs6956366	intron 1	4690870	C/G		33.2	0.748
*EGFR*	rs12718939	intron 1	4694689	A/G		32.7	0.994
*EGFR*	rs12668421	intron 1	4698546	T/A		27.1	0.943
*EGFR*	rs4947488	intron 1	4705924	A/T		27	0.876
*EGFR*	rs11766798	intron 1	4713688	A/G		26.3	0.891
*EGFR*	rs1024750	intron 1	4718100	A/G		20.7	0.906
*EGFR*	rs723527	intron 1	4724241	G/A		40.8	0.948
*EGFR*	rs10488140	intron 1	4727757	T/C		18.4	0.820
*EGFR*	rs10244108	intron 1	4741706	A/G		32.9	0.917
*EGFR*	rs12535226	intron 1	4745788	A/T		46.2	0.680
*EGFR*	rs4947490	intron 1	4749907	A/G		33.4	0.553
*EGFR*	rs6593205	intron 1	4758061	A/G		39.4	0.257
*EGFR*	rs2110290	intron 1	4762137	C/T		31.2	0.535
*EGFR*	rs759159	intron 1	4768769	T/G		38.3	0.620
*EGFR*	rs980653	intron 1	4775259	T/C		17.6	0.895
*EGFR*	rs13244925	intron 1	4781625	A/C		44.8	0.790
*EGFR*	rs13247687	intron 1	4791145	G/A		45.7	0.981
*EGFR*	rs12666347	Intron 1	4795299	T/A		33.5	0.948
*EGFR*	rs6964705	intron 1	4799006	C/A		45.7	0.993
*EGFR*	rs2072454	exon 4	4803717	T/C	synonymous	47.8	0.637
*EGFR*	rs4947986	intron 6	4811024	A/G		26.6	0.809
*EGFR*	rs4947987	intron 10	4814539	G/C		12	1.000
*EGFR*	rs2227983	exon 13	4818624	A/G	synonymous	23.6	0.417
*EGFR*	rs17337023	exon 16	4828243	A/T	synonymous	32.4	1.000
*EGFR*	rs9692301	intron 19	4833123	G/A		30.5	0.996
*EGFR*	rs846561	intron 20	4842077	C/T		24.7	0.928
*EGFR*	rs1554718	intron 20	4846332	T/C		42.4	0.963
*EGFR*	rs6970262	intron 21	4849132	A/G		36.5	0.978
*EGFR*	rs1140475	exon 23	4855786	T/C	synonymous	12.8	0.593
*EGFR*	rs2293347	exon 25	4858285	A/G	synonymous	9.5	0.960
*EGFR*	rs2280653	3′ near gene	4865463	C/T		18.2	0.990
*EGFR*	rs1107618	3′ near gene	4869652	A/G		20.4	0.83

SNP: Single nucleotide polymorphism.

*CALM3*: calmodulin 3, calcium-modulated protein 3.

*EGFR*: epidermal growth factor receptor.

*SMARCD1*: SWI/SNF-related matrix-associated actin-dependent regulator of chromatin subfamily d member 1.

MAF: minor allele frequency.

HWE: Hardy–Weinberg equilibrium.

UTR: untranslated region.

### Correlation between body compositions with potential covariates

As previously reported, age showed a statistically significant negative correlation with BMD at the three sites that were sampled in addition to lean mass levels (*P*<0.05). Age also demonstrated a statistically significant positive correlation with PTF levels (*P*<0.05). Tobacco use showed a statistically significant positive correlation with spine BMD, FNK BMD, TBL, TF and TL (*P*<0.05). However, as shown in **Table 4**, age and tobacco use were not closely correlated with these body compositions (all Pearson correlation coefficients were less than 0.5).

**Table 4:** Correlations between body compositions and potential covariates.

		SPINE BMD (n = 846)	Hip BMD (n = 846)	FNK BMD (n = 846)	Total body fat (n = 744)	Total body lean mass (n = 744)	PTBF (n = 744)	Trunk fat (n = 725)	Trunk lean (n = 725)	PTF (n = 725)
Age	Pearson correlation	−0.267	−0.247	−0.302	0.002	−0.123	0.039	0.022	−0.140	0.135
	Sig. (2-tailed)	<0.001**	<0.001**	<0.001**	0.965	0.001**	0.288	0.549	<0.001**	<0.001**
Tobacco use	Spearman correlation	0.078	0.057	0.086	0.032	0.079	0.017	0.093	0.117	0.067
	Sig. (2-tailed)	0.033*	0.121	0.020*	0.386	0.030*	0.639	0.012*	0.002**	0.070

BMD: bone mineral density; FNK: femoral neck; PTBF: percentage of total body fat; PTF: percentage of trunk fat.

*P<0.05,

**P<0.01.

### Significant associations between BMD at various sites and SNPs of the three genes

**Figure 1:**
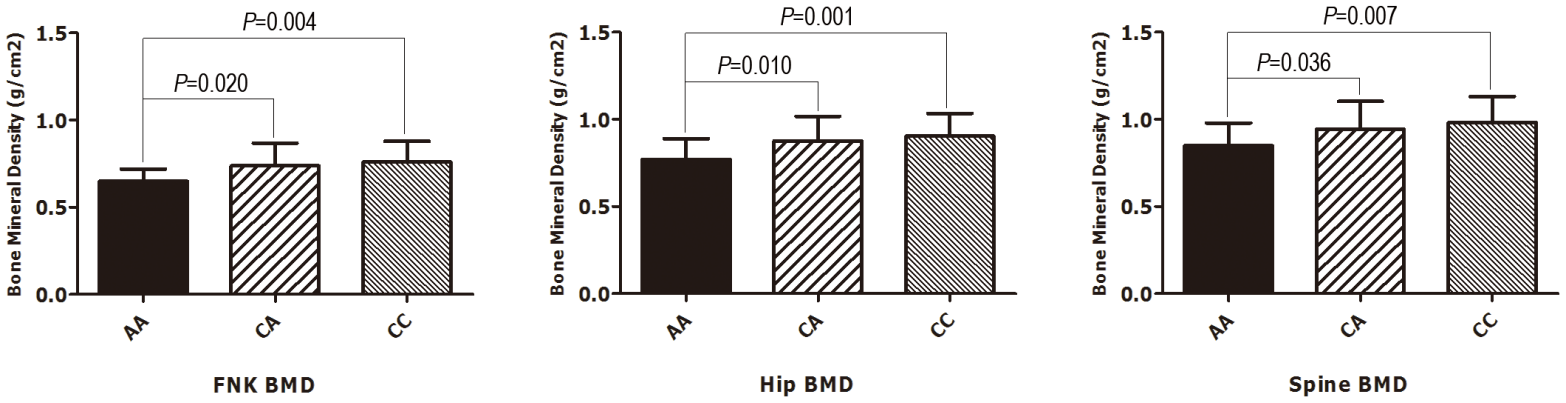
Bone mineral density at different sites associated with the *CALM3* SNP rs12461917 A/C polymorphism. FNK: femoral neck; BMD: bone mineral density.

**Figure 2:**
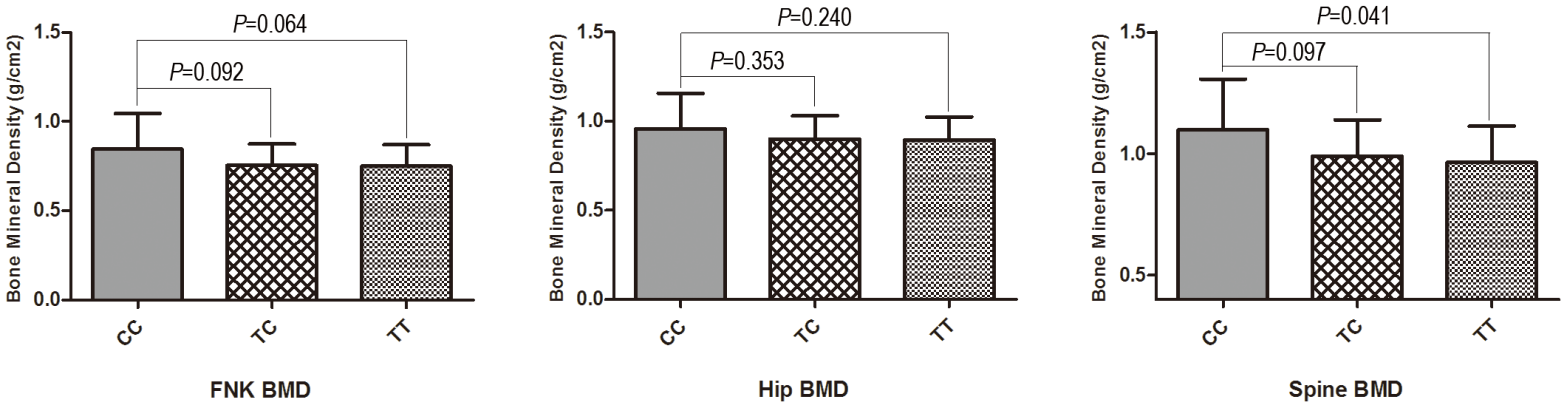
Bone mineral density at different sites associated with the *CALM3* SNP rs11083838 C/T polymorphism. FNK: femoral neck; BMD: bone mineral density.

As shown in figures 1 and 2, the FNK BMD, hip BMD and/or spine BMD levels of the carriers of the rs12461917 A/C polymorphism varied as follows: CC>CA>AA. Additionally, these BMD levels varied in the carriers of the rs11083838 C/T polymorphism as follows: CC>TC>TT.

We adjusted the levels of the phenotypes by significant covariates. Associations between the adjusted levels of body compositions and the 44 SNPs were tested by the Wald test that was implemented in PLINK. **Table 5** displays the results. A nominal significance level of 0.05 was set for each SNP. We observed that in the *CALM3* gene, the SNP rs12461917 in the 3′-flanking region displayed extremely significant associations with FNK BMD (*P* = 0.001), hip BMD (*P*<0.001) and spine BMD (*P* = 0.001). The SNP rs11083838 in the promoter of the gene also showed an extremely significant association with spine BMD (*P* = 0.009). In the EGFR gene, rs763317 in intron 1, rs4947986 in intron 6, rs9692301 in intron 19 and rs6970262 in intron 21 demonstrated marginally significant associations with the adjusted phenotypes. With respect to the significance for all SNPs at α = 0.05, after adjusting for multiple comparisons, rs12461917 remained significant (*P_adjusted_* = 0.033 for FNK BMD, *P_adjusted_* = 0.006 for hip BMD and *P_adjusted_* = 0.018 for spine BMD, respectively [data not shown]) as indicated by the asterisks in **Table 5**.

**Table 5:** Association analysis between the body compositions at various sites and the SNPs of three genes.

CHR	SNP	Spine BMD (n = 846)	Hip BMD (n = 846)	FNK BMD (n = 846)	Total body fat (n = 744)	Total body lean mass (n = 744)	PTBF (n = 744)	Trunk fat (n = 725)	Trunk lean mass (n = 725)	PTF (n = 725)
7	rs6969537	0.921	0.764	0.750	0.974	0.868	0.789	0.577	0.620	0.676
7	rs4947963	0.819	0.144	0.094	0.147	0.521	0.553	0.290	0.836	0.978
7	rs763317	0.714	0.057	0.048#	0.314	0.809	0.901	0.916	0.891	0.495
7	rs6956366	0.943	0.375	0.240	0.794	0.764	0.651	0.694	0.589	0.485
7	rs12718939	0.873	0.842	0.377	0.545	0.739	0.678	0.866	0.459	0.689
7	rs12668421	0.560	0.849	0.351	0.071	0.231	0.309	0.509	0.622	0.865
7	rs4947488	0.644	0.936	0.294	0.066	0.216	0.289	0.364	0.653	0.934
7	rs11766798	0.621	0.861	0.313	0.073	0.227	0.291	0.340	0.632	0.965
7	rs1024750	0.973	0.312	0.110	0.618	0.591	0.702	0.700	0.887	0.870
7	rs723527	0.483	0.440	0.181	0.324	0.794	0.676	0.823	0.634	0.475
7	rs10488140	0.727	0.778	0.803	0.805	0.944	0.935	0.738	0.911	0.954
7	rs10244108	0.886	0.590	0.640	0.181	0.544	0.579	0.761	0.526	0.578
7	rs12535226	0.955	0.391	0.922	0.654	0.445	0.720	0.331	0.294	0.270
7	rs4947490	0.476	0.695	0.726	0.752	0.591	0.513	0.922	0.728	0.704
7	rs6593205	0.155	0.289	0.167	0.862	0.518	0.951	0.474	0.532	0.870
7	rs2110290	0.994	0.600	0.782	0.378	0.405	0.460	0.693	0.271	0.860
7	rs759159	0.272	0.968	0.784	0.144	0.776	0.335	0.137	0.973	0.527
7	rs980653	0.928	0.845	0.676	0.769	0.535	0.960	0.960	0.450	0.862
7	rs13244925	0.914	0.303	0.325	0.254	0.858	0.407	0.721	0.764	0.935
7	rs13247687	0.589	0.483	0.683	0.819	0.591	0.736	0.698	0.244	0.332
7	rs12666347	0.871	0.612	0.927	0.658	0.695	0.941	0.993	0.750	0.681
7	rs6964705	0.865	0.803	0.430	0.808	0.585	0.729	0.600	0.243	0.281
7	rs2072454	0.240	0.578	0.677	0.153	0.103	0.111	0.072	0.141	0.018#
7	rs4947986	0.832	0.919	0.882	0.184	0.026	0.064	0.225	0.085	0.032#
7	rs4947987	0.119	0.336	0.671	0.470	0.718	0.368	0.462	0.685	0.567
7	rs2227983	0.437	0.386	0.571	0.546	0.555	0.621	0.459	0.191	0.461
7	rs17337023	0.712	0.839	0.982	0.353	0.687	0.540	0.595	0.259	0.667
7	rs9692301	0.580	0.733	0.665	0.120	0.037	0.066	0.488	0.075	0.125
7	rs845561	0.560	0.520	0.963	0.498	0.992	0.472	0.099	0.695	0.176
7	rs1554718	0.867	0.607	0.429	0.280	0.394	0.183	0.552	0.553	0.917
7	rs6970262	0.870	0.926	0.877	0.332	0.096	0.195	0.070	0.330	0.029#
7	rs1140475	0.595	0.547	0.454	0.204	0.115	0.214	0.200	0.098	0.083
7	rs2293347	0.218	0.837	0.713	0.313	0.626	0.542	0.269	0.084	0.668
7	rs2280653	0.623	0.988	0.607	0.804	0.980	0.903	0.379	0.264	0.737
7	rs1107618	0.999	0.743	0.790	0.373	0.539	0.492	0.203	0.193	0.729
12	rs11169270	1.000	0.424	0.640	0.235	0.702	0.415	0.629	0.353	0.534
12	rs7139363	0.895	0.052	0.409	0.349	0.263	0.487	0.801	0.067	0.768
12	rs836172	0.857	0.411	0.581	0.769	0.512	0.956	0.662	0.638	0.909
12	rs836177	0.693	0.386	0.569	0.691	0.577	0.883	0.631	0.802	0.858
19	rs11083838	0.009##	0.183	0.180	0.375	0.346	0.277	0.155	0.126	0.080
19	rs7259810	0.767	0.985	0.888	0.666	0.793	0.580	0.755	0.369	0.940
19	rs4380146	0.424	0.130	0.140	0.729	0.521	0.711	0.315	0.736	0.319
19	rs10113	0.154	0.559	0.727	0.303	0.889	0.256	0.312	0.615	0.463
19	rs12461917	0.001^##^ *	0.001^##**^	0.001^##^ *	0.268	0.696	0.424	0.926	0.687	0.924

CHR: chromosome; SNP: Single nucleotide polymorphism; BMD: bone mineral density; FNK: femoral neck; PTBF: percentage of total body fat; PTF: percentage of trunk fat.

All *P* values were unadjusted;

# *P*<0.05,

## *P*<0.01,

*P, **P were adjusted using Max (T) in PLINK to correct for multiple testing, *P<0.05, **P<0.01.

For the two significant SNPs (rs12461917 and rs11083838) of *CALM3*, we compared the raw BMD levels at the spine, hip and femoral neck in association with the three genotypic variants of rs12461917 and rs11083838 according to a one-way ANOVA. The A/C polymorphism at rs12461917 was associated with significant variations in BMD at all three sites (**Fig 1**). The only differences in the spine BMD that were found to be statistically significant were observed in association with the C/T polymorphism of rs11083838, particularly between the CC and TT genotypes (*P* = 0.041, **Fig 2**).

## Discussion

The current study reveals the associations of the polymorphisms of the three candidate genes with body composition levels in postmenopausal Caucasian women. Adjusting for smoking status and age, we found statistically significant associations between the rs12461917 A/C polymorphism of *CALM3* and femoral neck BMD, hip BMD and spine BMD. The BMD levels at these three sites consistently varied in accordance with the polymorphisms from CC>CA>AA. The rs11083838 C/T polymorphisms of *CALM3* were associated with spine BMD differences. Spine BMD levels varied in accordance with the polymorphisms from CC>TC>TT; however, only the BMD difference between CC and TT was statistically significant. More impressively, these statistically significant BMD differences were associated with the *CALM3* polymorphisms even though only 5 candidate SNPs were assessed. These results demonstrate that mutations of the *CALM3* gene do indeed affect BMD levels in postmenopausal Caucasian women. Our findings are in accordance with current knowledge regarding the role of CALM3 as a vital regulator of osteoclastic differentiation, functional bone resorption and apoptosis [14].

The *CALM* gene series, including *CALM1*, *CALM2* and *CALM3*, encode completely identical CaM proteins, which are found in all eukaryotic cells and act as common, highly conserved Ca^2+^ sensors that regulate various types of cellular pathways. Although the three *CALM* genes encode identical amino acids, their coding sequences differ markedly in their nucleotide compositions. The *CALM3* gene is actively transcribed at 5-fold greater levels compared with the other two genes [34], highlighting the importance of this particular gene to CaM functioning. To date, few mutation sites are known in the exons of the three genes, which are associated with low mutation frequencies. The data pertaining to the *CALM* genes are mainly associated with the 5′- or 3′-flanking regions. A previous study [35] reported that the −34T > A *CALM3* polymorphism in the promoter (5′-flanking region) is a potential modifier for familial hypertrophic cardiomyopathy because it affects the expression levels of *CALM3*. Our study found that BMD was associated with both rs11083838 polymorphisms in the 5′-flanking region of *CALM3* and rs12461917 polymorphisms in the 3′-flanking region. These results show that both the 5′- and 3′-flanking regions may play important roles in the regulation of CALM3 function. Although the significance of the 5′ flanking region to CALM3 function was previously validated [35], to our knowledge, this is the first report of the 3′ flanking region being implicated in CaM functionality, thus opening avenues to future research. The manner by which the polymorphisms of *CALM3* affect BMD levels at the different sites is unknown and will require further study.

EGFR plays a known role in both osteoclast and osteoblast function [9], [10] and has also been shown to interact directly with CaM [12], [13]. SMARCD1 acts as an important regulator of mesenchymal stem cells, from which both bone and fat are derived [36], and as a known mediator of lipid metabolism, which may indirectly influence BMD [18], [36]. Furthermore, as previously mentioned, a recently demonstrated novel interaction of SMARCD1 with the vitamin D receptor also may underlie the potential involvement of this protein in bone metabolism [19]. Despite the plausible biological mechanisms for both SMARCD1 and EGFR participation in bone processing, our study shows no statistically significant association between polymorphisms in *SMARCD1* and BMD and only a marginally significant association between *EGFR* and BMD. It is possible that other *SMARCD1/EGFR* polymorphisms that were not included in this study may show more significant associations with BMD levels; therefore, a larger and perhaps more inclusive study, such as a study of postmenopausal women of multiple races, may be beneficial in this regard.

None of the gene polymorphisms that were studied in this paper, even those of *CALM3*, were found to be associated with any other body composition levels. It is possible that the effects of other potential unidentified factors, such as socioeconomic status (SES) and diet, may mask the effects of the variances in these target genes on body composition given that age and smoking were the only two covariates that were adjusted for.

This study, although limited in scope due to its focus on postmenopausal Caucasian woman, provides multiple opportunities for further investigation. A study of the impact of *CALM3* and other target genes on BMD across several race/ethnicity groups may be informative because racial differences in BMD have been well established in the literature [37], [38]. This study demonstrates that genetic polymorphisms in genes that are involved in bone metabolism may impact BMD at least in Caucasian women. Further research is required to elucidate whether these polymorphisms and others that are yet to be discovered may also partially underlie the racial differences that are observed in BMD.
